# Characterization of high affinity IgM and IgG monoclonal antibodies against norovirus variants GII.4 and GII.17

**DOI:** 10.1002/pro.70522

**Published:** 2026-03-12

**Authors:** Jumpei Tagawa, Saeko Yanaka, Yuri Kato, Akitsu Masuda, Jae Man Lee, Akinobu Senoo, Kosuke Oyama, Takayuki Uchihashi, Motohiro Nishida, Takahiro Kusakabe, Jose M. M. Caaveiro

**Affiliations:** ^1^ Graduate School of Pharmaceutical Sciences Kyushu University Fukuoka Japan; ^2^ Laboratory for Materials and Structures, Institute of Integrated research Institute of Science Tokyo Kanagawa Japan; ^3^ Laboratory of Creative Science for Insect Industries Kyushu University Graduate School of Bioresource and Bioenvironmental Sciences Fukuoka Japan; ^4^ Department of Biological Science and Technology Tokyo University of Science Tokyo Japan; ^5^ Graduate School of Sciences Nagoya University Nagoya Aichi Japan; ^6^ Laboratory of Insect Genome Science Kyushu University Graduate School of Bioresource and Bioenvironmental Sciences Fukuoka Japan

**Keywords:** avidity, immunoglobulin G, immunoglobulin M, norovirus

## Abstract

Human noroviruses are a leading cause of acute gastroenteritis worldwide, yet the molecular principles governing antibody recognition of their highly repetitive capsid remain poorly understood. Here, we immunized mice with virus‐like particles (VLPs) from the pandemic GII.4 strain and the emergent GII.17 strain, generating monoclonal IgM and IgG antibodies via hybridoma technology. High‐speed atomic force microscopy visualized IgM antibodies scanning and engaging multiple protruding (P) domains on intact VLPs. Surface plasmon resonance (SPR) analyses of engineered antibodies with identical Fab sequences but different valencies revealed that, unlike monovalent IgG, multivalent IgM exhibits dramatic affinity gains—up to 100‐fold—as antigen density increases. This avidity‐driven enhancement arises from the dense, repetitive P‐domain architecture of the norovirus capsid, enabling IgM to achieve high functional affinity despite modest intrinsic Fab binding. Our findings define how antibody valency and epitope organization cooperate to boost viral recognition, offering a mechanistic framework for designing next‐generation vaccines and antiviral antibodies that harness multivalent engagement.

## 1 INTRODUCTION

Human norovirus is recognized as the leading cause of sporadic and epidemic viral gastroenteritis (Patel et al., 2008; Robilotti et al., 2015) and is estimated to be responsible for approximately 699 million norovirus infections, more than 1 million hospitalizations, and 219,000 deaths worldwide, resulting in a social cost of $60 billion annually (Bányai et al., 2018; Bartsch et al., 2016). Thus, the global burden caused by norovirus is substantial and requires sensitive and accurate diagnosis and effective therapeutics and vaccination.

Based on the primary sequence of VP1 protein of the capsid, noroviruses have been phylogenetically classified into at least 10 gene groups (GI‐GX) and further subdivided into 49 genotypes (e.g., GII.4) (Chhabra et al., 2019). Among the norovirus genotypes, GII.4 strains represent the dominant group of human infectious noroviruses worldwide (55%–85%) and has been the predominant genotype for more than 20 years (Cannon et al., 2021; Desai et al., 2012). Significant attention has thus been focused on the genotype of GII.4 strains in human populations (Lindesmith et al., 2008; Tan & Jiang, 2005). However, the emergence of a new genotype, strain GII.17, in the winter of 2014–2015, caught the world's attention and surpassed strain GII.4 as the main cause of norovirus outbreaks in several countries in late 2014 (Chan et al., 2015; De Graaf et al., 2015). Genomic nucleotide sequences of structural proteins can differ by more than 50% between gene groups (Pletneva et al., 2001), and antibodies produced by GII.4 strains generally lack cross‐reactivity to GII.17 strains (Dai et al., 2017; Du et al., 2021). Because genetic diversity in structural proteins also causes changes in antigenic properties, it is essential to understand the human immune response to infection via human noroviruses and antigenic variation among circulating human norovirus strains.

Noroviruses are non‐enveloped RNA viruses whose outer surface is covered by a capsid protein composed of 180 monomeric units. The components of the capsid are the capsid protein VP1 and the minor structural protein VP2 (Vongpunsawad et al., 2013); VP1 comprises two domains, a highly conserved shell (S) domain and a more variable protruding (P) domain (Prasad et al., 1999). In the human immune system, infection with noroviruses results in the production of neutralizing antibodies against these components.

The complexity of human norovirus cross‐reactivity and neutralization by human antibodies is not yet fully realized. A number of studies have evaluated the existence of a human polyclonal immune response to human norovirus (Czakó et al., 2015; Lindesmith et al., 2005, 2019). Studies analyzing mAbs isolated from norovirus‐infected humans and mice using hybridoma technology have primarily isolated IgG‐type antibodies and have also identified IgA and IgM‐type antibodies (Alvarado et al., 2018; Gray et al., 1994; Sapparapu et al., 2016; Tanaka et al., 2006). Previous epitope analysis of norovirus antibodies have identified neutralizing epitopes on norovirus capsids primarily against the protruding 2 (P2) domain, but also conserved epitopes in inaccessible regions of the viral capsid (Lindesmith et al., 2012; Van Loben Sels & Green, 2019). Indeed, antigen mapping studies using strain‐specific P and S domains suggested that some of the highly cross‐reactive mAbs bind to the S domain (Alvarado et al., 2021; Parra et al., 2013). Epitope analysis of IgGs against GII.4 strains has advanced because of the variety of their antibodies. In contrast, epitope mapping of mAbs against GII.17 is limited because of the few examples of GII.17‐specific mAbs (Strother et al., 2023; Yi et al., 2021). Moreover, detailed studies of anti‐norovirus antibodies of the IgM class are even more limited. Although avidity effects of multivalent antibodies have been conceptually recognized, quantitative analyses dissecting the impact of antigen density and antibody valency remain scarce. To our knowledge, no previous study has systematically compared antibodies of identical Fab sequence but different valencies under controlled antigen density conditions for norovirus or other viruses. Because VLP are complex entities with high density of P‐domain, which is the major epitope of norovirus antibodies, IgM displaying 10 or 12 antigen‐binding domains could bind to those with high affinity due to their multivalency (Keyt et al., 2020; Oostindie et al., 2022; Wibroe et al., 2014).

In this study, we obtained hybridoma cells producing monoclonal antibodies that specifically recognized strains GII.4 or GII.17. Antibodies of the IgG and IgM class were obtained, and the molecular basis of their efficient binding to norovirus was evaluated by complementary techniques.

## 2 RESULTS AND DISCUSSION

### 2.1 Selection of hybridoma cells secreting antibodies against norovirus

Hybridoma cells were generated in triplicate, thus dividing the selection of hybridoma cells into three groups. Hybridomas producing antibodies specific to GII.4 and GII.17 were selected by a two‐step selection process. The first and second groups of hybridoma cells were selected by ELISA using norovirus VLPs, and the third group was selected by ELISA using norovirus P‐domain (Supplementary Figure 1a).

In the primary screening, wells with absorbance more than three times higher than the blank were considered positive in ELISA. Hybridoma cells generated from spleen cells derived from 60 mice (group 1, group 2 and group 3 consisted of 18, 18 and 24 animals, respectively), were seeded to 56 96‐well plates (group 1, group 2 and group 3 corresponded to 30, 18 and 8 plates, respectively). Hybridoma cells from 141 different wells (group 1, group 2, and group 3 gave 80, 40, and 21 positives, respectively). ELISA positives corresponded to antigens containing the sequence of the GII.4 strain or to the sequence of the GII.17 strain (Supplementary Figure 1b).

In the secondary screening, additional ELISA was performed with the hybridoma cells obtained from the 141 wells to exclude nonspecific binding. To that end we employed the norovirus unrelated protein ovalbumin. After this step, 16 different samples of hybridoma cells were selected. These cells corresponded to group 1, group 2, and group 3 containing 7, 6, and 3 positive clones, respectively. These 16 hybridoma cell samples were subjected to the limiting dilution method yielding seven unique clones of cells producing monoclonal antibodies that we termed according to their position in the plate in the experiment above (12D7, 14A10, 3G6, 13G1, 14C2, 18F9, and 5H8) (Supplementary Figure 1c).

### 2.2 Identification of antibody isotypes and their purification

Antibody classes and subclasses were determined for the seven different mAbs‐producing hybridoma cells using a commercial kit (Table 1). Of the seven antibodies examined, five were found to belong to the IgM class and two to the IgG class. To purify the IgG‐class antibodies (14C2 and 5H8), we employed Protein G affinity chromatography. For the IgM antibodies (which are not retained by protein A or protein G), we had to consider alternative methods for purification. Protein L is known to specifically bind to the light chain with a known sequence (Paloni & Cavallotti, 2017). We conducted sequence analysis of the light chains of the obtained IgM and revealed that sequence motifs important for protein L binding were preserved for two of them (12D7 and 3G6). For these two IgM clones, protein L was used for purification. The other IgMs were purified using an IgM affinity column, although it is worth noting that this column displayed lower specificity toward IgM than Protein A or Protein L showed for IgG. Therefore, 13G1 and 18F9 exhibited slightly lower purity in the SDS‐PAGE (Supplementary Figure 2a). Secondary purification was then performed using size exclusion chromatography (SEC), confirming elution peaks at around 150 kDa for antibodies of the IgG class, and peaks around 900 kDa for the antibodies of the IgM class (Supplementary Figure 2b). Analysis by SDS‐PAGE under reducing conditions confirmed the purification of the IgG‐type antibody and IgM‐type antibody. All the samples were prepared with a satisfactory level of purity (Supplementary Figure 2a).

**TABLE 1:** Antibody isotypes.

Clone name	Mouse isotyping kit result
12D7	IgM
14A10	IgM
3G6	IgM
13G1	IgM
14C2	IgG2b
18F9	IgM
5H8	IgG3

### 2.3 Evaluation of binding ability of purified antibodies to norovirus VLPs

To evaluate the binding ability of seven types of anti‐norovirus antibodies purified by affinity chromatography and SEC, binding assays using ELISA were conducted. Consistent with the results obtained from screening, all antibodies were confirmed to bind to norovirus VLPs, with some antibodies being specific to GII.4 norovirus VLPs (12D7, 14A10, 3G6, 13G1) and some antibodies being specific to GII.17 norovirus VLPs (14C2, 18F9, 5H8) (Supplementary Figure 3).

### 2.4 Visualization of binding to VLPs by high‐speed atomic force microscopy (HS‐AFM)

**FIGURE 1:**
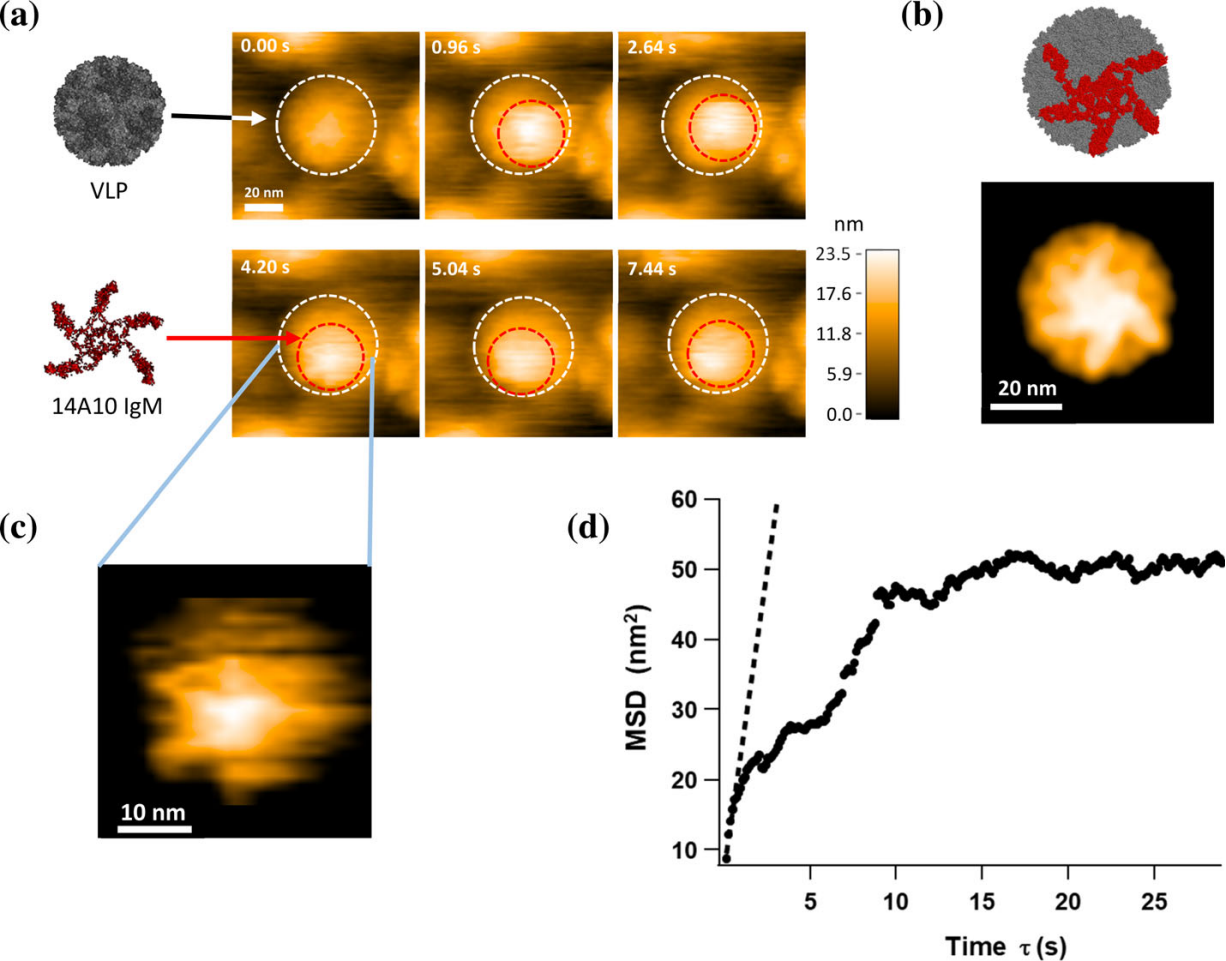
Dynamic interaction of 14A10 IgM with norovirus VLPs observed by HS‐AFM. (a) Representative snapshots of 14A10 IgM binding to a norovirus GII.4 VLP and 14A10 IgM are indicated by white and red dotted circles, respectively. The color bar indicates the height in nanometers. Snapshots were taken between 54.84 and 67.08 s from the start of the observation, showing the lateral movement of the IgM molecule on the VLP surface. (b) Structural model of the pentameric IgM antibody bound to the VLP surface (top) and the corresponding pseudo‐AFM image (bottom). The pseudo‐AFM image reveals a star‐shaped morphology with radiating protrusions, exhibiting a striking similarity to the experimental observations shown in (a). (c) Magnified and filtered image of the bound IgM. The image is a magnified view of the IgM molecule captured at 4.2 s in (a), processed with a bandpass filter (spatial frequency: 2.5 nm–10 nm) to enhance structural contrast. Five prominent “legs” (representing the Fab or Fc domains) of the pentameric IgM are discernible, suggesting multi‐valent tethering to the VLP surface. (d) Ensemble‐averaged mean square displacement (MSD) plot of 14A10 IgM moving on the VLP surface (*n* = 28). The red dashed line represents the linear fit to the initial slope (*τ* = 0.12–0.60 s) used to calculate the diffusion coefficient (*D*~8.5 nm^2^/s). The plateau at approximately 57 nm^2^ indicates that the IgM molecule undergoes confined diffusion within a localized area of the VLP surface.

Using HS‐AFM, the interaction between IgM antibodies and norovirus VLPs was visualized in real‐time at the single‐molecule level. HS‐AFM images of VLPs showed spherical particles with a diameter of approximately 40 nm. Upon the addition of IgM antibody 14A10, dynamic binding of IgM antibodies to the VLP surface was clearly observed (Figure 1a). The morphology of the bound IgM in these snapshots characterized by a central body with extending domains is basically consistent with a structural model and corresponding pseudo‐AFM image of a pentameric IgM molecule docked onto the curved VLP surface (Figure 1b). Notably, the bound IgM antibodies did not remain stationary but continuously moved across the VLP surface during the observation period (Movie S1). Typically, only one IgM molecule was observed per VLP. Given the molecular size of IgM (approximately 30 nm in diameter) and the projected surface area of the VLP hemisphere, a single IgM molecule likely occupies most of the accessible upper surface of the VLP, sterically hindering the attachment of additional antibodies.

To further examine the structure of the bound antibody, we focused on the IgM molecule at 4.2 s in Figure 1a. A magnified image significantly enhanced the contrast of the IgM, allowing for the visualization of five prominent “legs” (likely representing the Fab or Fc domains) extending from the central part of the molecule (Figure 1c). This structural detail suggests that the IgM molecule tethers to the VLP surface through multiple domains. This experimental observation of the pentameric arrangement directly supports the multivalent tethering mode depicted in the structural model (Figure 1b).

To quantify this movement, the centroid position of the IgM molecule was tracked (Movie S2) and the resulting trajectories were used for mean square displacement (MSD) analysis. To characterize the dynamics of 14A10 IgM on the VLP surface, ensemble‐averaged MSD analysis was performed using 28 independent trajectories. The MSD plot exhibited a characteristic plateau at approximately 57 nm^2^, identifying the movement as confined diffusion (Figure 1d). The initial diffusion coefficient (*D*) was estimated to be 8.5 nm^2^/s from the slope of the MSD curve at short time lags (*τ* = 0.12–0.60 s), confirming that the IgM molecule actively scans the surface. The estimated area of the exploration patch was approximately 360 nm^2^ (derived from the MSD plateau, where the patch radius a~11 nm). Considering that the observable projected area of the VLP is approximately 1260 nm^2^, the exploration area is restricted to less than 30% of the available surface.

This localized scanning behavior, combined with the remarkably high binding stability, where 14 out of 31 events (approx. 45%) lasted longer than the 90 s observation window, provides a physical basis for the high functional avidity of 14A10 IgM. These findings suggest a “sliding” mechanism where the IgM remains tethered to the capsid via multiple Fab‐epitope interactions while dynamic scanning ensures continuous occupancy of the viral surface. This robust attachment and physical shielding of the VLP surface likely contribute to the potent inhibitory activity of this antibody.

### 2.5 Analysis of the effect of antigen density of norovirus P‐domain on the binding of anti‐norovirus antibodies

**FIGURE 2:**
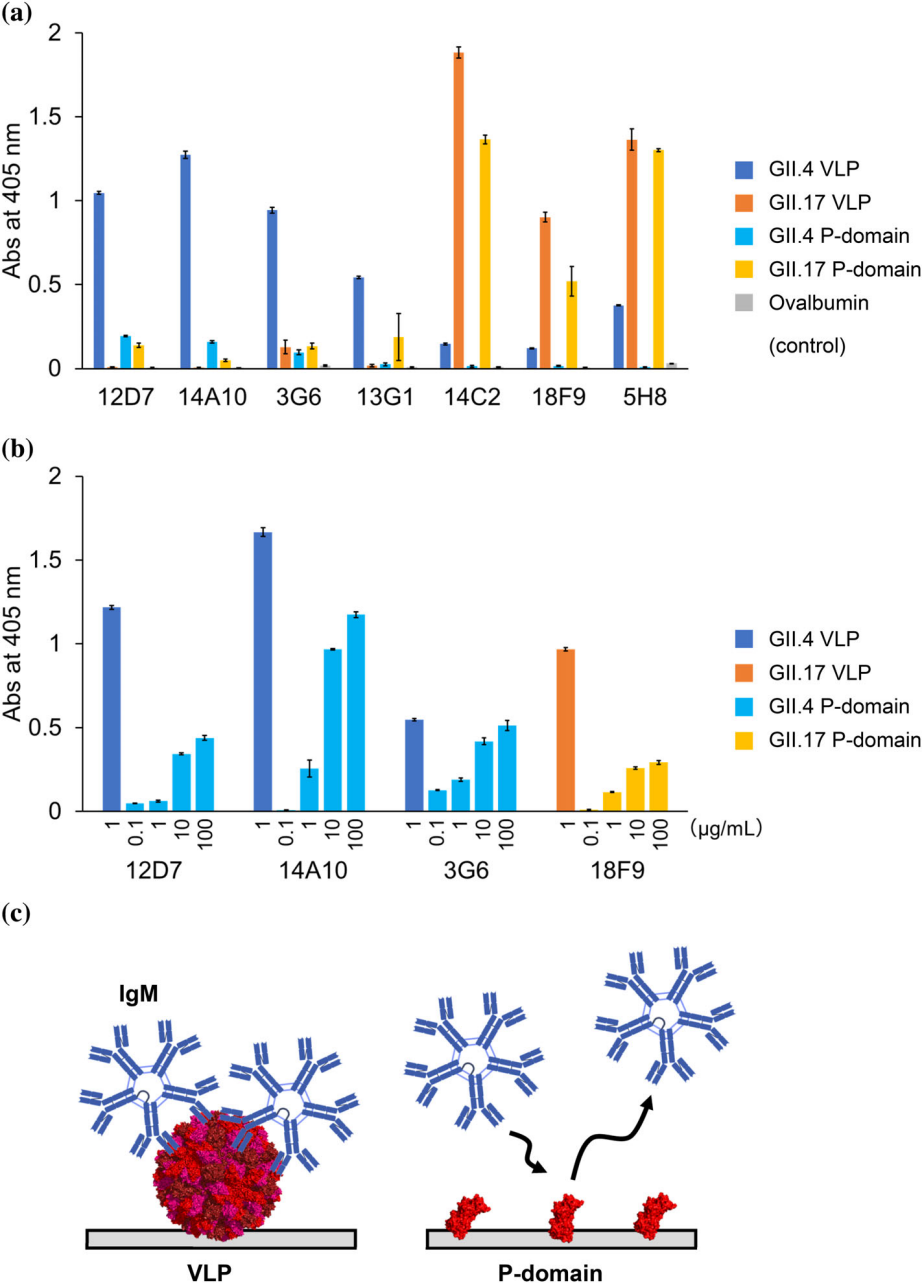
Evaluation of binding specificity of anti‐norovirus antibodies by ELISA. (a) Indirect ELISA was used to evaluate the binding of seven mouse mAbs to GII.4 VLP, GII.17 VLP, GII.4 P‐domain, and GII.17 P‐domain, respectively. Error bars indicate standard deviation (*n* = 3). (b) Indirect ELISA was used to evaluate the binding of four anti‐norovirus IgM to VLP of GII.4 strain, P‐domain of GII.4 strain, VLP of GII.17 strain and P‐domain of GII.17 strain, respectively, with varying immobilization density of norovirus P‐domain. Error bars indicate standard deviation (*n* = 3). (c) Schematic model illustrating the proposed binding of IgM to VLP and the P‐domain immobilized on an ELISA plate.

To evaluate whether the obtained monoclonal antibodies possess epitopes on the P‐domain of norovirus GII.4 and GII.17 strains and how the antigen density affects their binding ability, we prepared P‐domain of norovirus GII.4 and GII.17 strains (Supplementary Figure 4) and binding assays using ELISA were conducted (Figure 2). First, VLPs and P‐domains were immobilized to have equivalent mass numbers, and the assay confirmed binding of four types of IgM antibodies (12D7, 14A10, 3G6, 18F9) and two types of IgG antibodies (14C2, 5H8) to the recombinant P‐domain (Figure 2a). Thus, this experiment clarified that these six antibodies recognize epitopes on the P‐domain region of the VP1 protein. Regarding antibody 13G1 of the IgM class, binding to norovirus VLPs was confirmed, but no binding to the P‐domain was observed. Interestingly, antibody 13G1 consistently bound to intact VLPs but showed no detectable interaction with the isolated P‐domain, even after repeated ELISA assays under the tested conditions. This observation suggests that 13G1 either recognizes an epitope located outside the P‐domain, such as within the shell (S) domain, or requires the structural context and high‐density presentation of epitopes provided by the intact VLP. These possibilities highlight the complexity of norovirus antigenicity and warrant further structural investigation.

When comparing the level of binding of the antibodies, it was clear that the binding strength of the monoclonal antibodies to VLPs or to P‐domains differed significantly, showing that IgM binds to VLP more effectively than to the isolated P‐domain, even if the same concentration of epitope units were immobilized in the ELISA assay. This effect was especially obvious when comparing IgM and IgG antibodies (except for 18F9). For example, the values of absorbance of IgM antibodies 12D7 and 14A10 after binding to VLP were more than five‐fold greater than that of samples bound to the purified P‐domain (the absorbance values resulting from the binding of 12D7 to GII.4 VLP and to purified P domain were 1.05 and 0.19, respectively; also, the absorbance values resulting from the binding of 14A10 to GII.4 VLP and to purified P‐domain were 1.27 and 0.16, respectively). In contrast, the absorbance resulting from the binding of IgG antibodies 14C2 and 5H8 to VLP particles or to the P‐domain did not change all that much (the absorbance resulting from the binding of 14C2 to GII.17 VLP and to the purified P‐domain were 1.88 and 1.37, respectively; similarly, for 5H8 the values of absorbance resulting from the binding to GII.17 VLP and to P‐protein were 1.36 and 1.30, respectively) (Figure 2a).

Since norovirus VLPs have a capsid structure composed of 180 units of P‐domain, P‐domain molecules are densely packed on the immobilized VLP particles. In contrast, norovirus P‐domain monomers are uniformly immobilized on the surface of the ELISA plates. It is thus expected that the average distance between immobilized antigens will be greater than that of the very densely packed VP1 molecules in the VLP particles. Therefore, we hypothesized that the difference in antigen density might affect the binding of IgM antibodies (Figure 2c). Indeed, it is said that the binding strength of an antibody is affected by the valency (i.e., the number of Fab moieties), and several assays have been developed (Liu et al., 2020). Hence, we hypothesize that IgM with 10 Fabs in one molecule is expected to have a higher binding ability in areas with high epitope density. In fact, HS‐AFM clearly visualizes that IgM is using the 10 Fabs effectively to interact with norovirus VLPs, which consist of 180 tightly packed P‐domains.

To clarify the impact of epitope density on the surface of the ELISA plate, we investigated whether the amount of antibody binding changes when controlling the antigen immobilization level of the P‐domain. The IgM antibodies 12D7, 14A10, 3G6, and 18F9 showed an increase in absorbance of the P‐domain with increasing immobilization level of the P‐domain (Figure 2b). In particular, when the concentration of P‐domain incubated on the ELISA plate increased from 1 to 10 μg/mL, there was a significant increase in absorbance, suggesting that the avidity effect of IgM was not strongly manifested at immobilization densities below 1 μg/mL, whereas at immobilization densities above 10 μg/mL, the avidity effect of IgM was strongly manifested. Conversely, compared to the absorbance of VLPs at an immobilization amount of 1 μg/mL, the absorbance of P‐domain at an immobilization amount of 100 μg/mL was lower than that of VLPs, implying that due to its structural nature, VLP has a very high epitope density, allowing IgM to bind efficiently.

The downside of ELISA assay is that the immobilization level cannot be quantified. More precise control of immobilization level is needed to discuss the effect of density on the avidity effect of antibody. To further elucidate the efficacy of avidity effects based on Fab valency, we considered analyzing the impact of valency on affinity using surface plasmon resonance (SPR). Additionally, to closely examine the effects of valency, we planned to create a series of antibodies with the same Fab but different valency. To select suitable IgM and IgG for such analyses, we first evaluated the affinities of the obtained antibody groups using SPR.

### 2.6 Exploration of the impact of antigen immobilization level and binding valency on affinity

**FIGURE 3:**
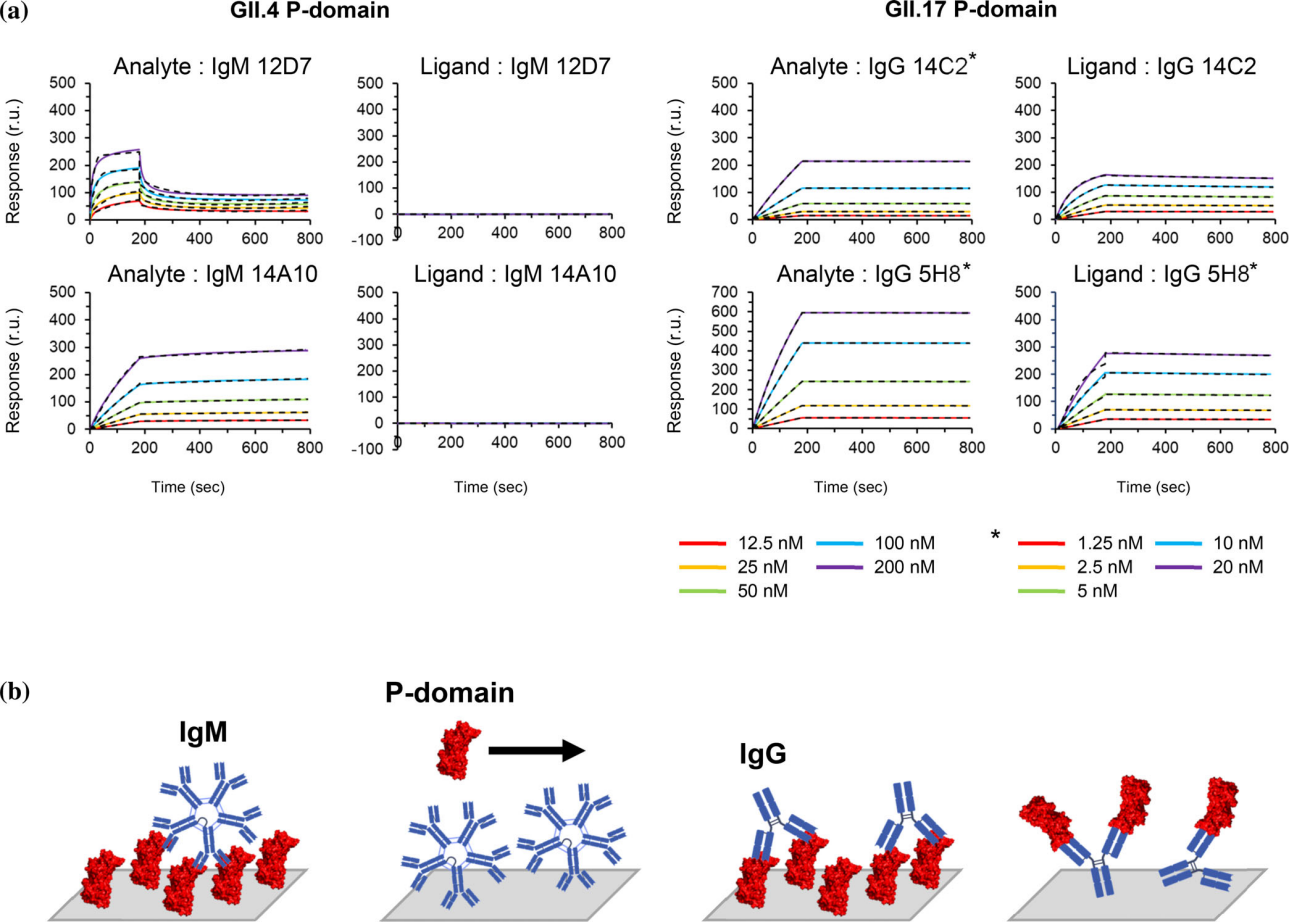
Interaction analysis of anti‐norovirus antibodies with norovirus P‐domain by SPR. (a) Binding of anti‐norovirus antibodies to norovirus P‐domain was evaluated by SPR. The solid line shows the obtained sensorgrams and the fitting is shown as a dashed line. (b) Models of SPR interaction between anti‐norovirus antibodies and norovirus P‐domain. From left: IgM and immobilized P‐domain, P‐domain and immobilized IgM, IgG and immobilized P‐domain, P‐domain and immobilized IgG.

The binding affinity of seven types of monoclonal antibodies to P‐domains of norovirus GII.4 and GII.17 strains was determined using SPR (Supplementary Figure 5). It was revealed that antibody 14A10 among IgM antibodies and antibody 14C2 among IgG antibodies had high binding affinities. Thus, 14A10 and 14C2 were selected as representative of IgM and IgG antibodies, respectively, for analyzing the effect of valency (Figure 3, Table 2).

**TABLE 2:** Kinetic analysis for the interaction of anti‐norovirus antibodies with immobilized P‐domain and interaction of P‐domain with immobilized anti‐norovirus antibodies.

Ligand	Analyte	*K*_on_ (/Ms)	*k*_off_ (/s)	*k*_D_ (M)
GII.4 P‐domain	12D7 IgM	1.85 × 10^6^	1.71 × 10^−2^	9.21 × 10^−9^
12D7 IgM	GII.4 P‐domain	N.D	N.D	N.D
GII.4 P‐domain	14A10 IgM	3.31 × 10^4^	1.06 × 10^−5^	3.21 × 10^−10^
14A10 IgM	GII.4 P‐domain	N.D	N.D	N.D
GII.17 P‐domain	14C2 IgG	1.16 × 10^5^	1.07 × 10^−7^	9.21 × 10^−13^
14C2 IgG	GII.17 P‐domain	8.29 × 10^4^	1.29 × 10^−4^	1.55 × 10^−9^
GII.17 P‐domain	5H8 IgG	2.11 × 10^5^	1.09 × 10^−5^	5.15 × 10^−11^
5H8 IgG	GII.17 P‐domain	7.83 × 10^5^	6.37 × 10^−5^	8.13 × 10^−11^

To quantitatively analyze the impact of valency on affinity using SPR, a series of antibodies with the same type of Fab moiety but different numbers of them (different valences) were prepared and their purification purity confirmed (Supplementary Figure 6). The IgM antibody 14A10 was established as the model for IgM antibodies, and the Fab region of 14A10 was designated as a monovalent antibody and termed 1‐arm IgM. A bivalent IgG class switch variant of 14A10, with the Fab region combined with the Fc region of the anti‐HER2 human IgG antibody trastuzumab, was termed 2‐arm IgM, while the original 14A10 IgM was termed 10‐arm IgM. Additionally, the Fab region of the IgG 14C2 antibody was prepared and termed 1‐arm IgG antibody.

**FIGURE 4:**
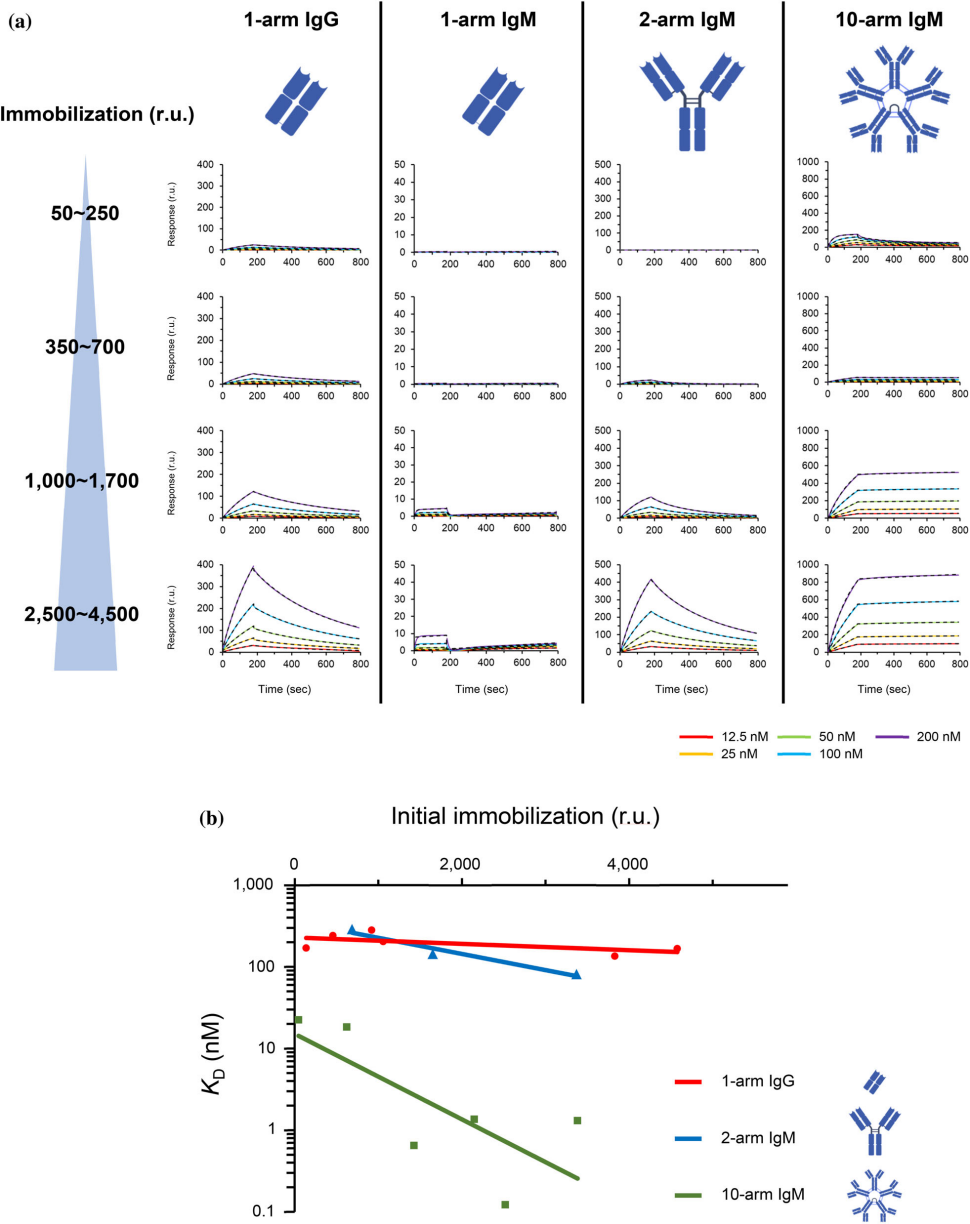
Interaction analysis of norovirus P‐domain with a group of antibodies with different binding titers at different immobilization densities by SPR. (a) Binding of 1‐arm IgG to the P‐domain of norovirus GII.17, 1‐arm IgM, 2‐arm IgM, and 10‐arm IgM to the P‐domain of norovirus GII.4 were evaluated by SPR. The solid line shows the obtained sensorgrams and the fitting is shown as a dashed line. (b) The amount of P‐domain immobilized versus binding affinity in SPR is plotted. 1‐arm IgG is indicated by red circles, 2‐arm IgM by blue triangles, and 10‐arm IgM by green qualifications. Exponential approximation curves were obtained from the plotted points, respectively.

The interactions of 1‐arm IgM, 2‐arm IgM, 10‐arm IgM, and 1‐arm IgG with the norovirus P‐domain were analyzed by varying the antigen immobilization level, which influences the density of epitope on SPR surface (Figure 4a, Table 3). The obtained sensorgrams were initially compared at the same immobilization levels across antibodies. That is, by examining the results horizontally (i.e., at approximately constant antigen immobilization level), it was revealed that as the valency increased from 1‐arm IgM to 2‐arm IgM and to 10‐arm IgM, the affinity increased (the affinity of 1‐arm IgM at 3274RU was N.D., the affinity of 2‐arm IgM at 3372 RU was 8.15 × 10^−8^ M, and the affinity of 10‐arm IgM at 2520RU was 1.22 × 10^−10^ M, which is 1500 times higher than that of 2‐arm IgM). Thus, although the antigen binding ability of each Fab was weak, IgM antibody 14A10 could bind to the antigen due to the avidity effect. Furthermore, the 1‐arm IgG showed a significantly greater binding ability than that of the 1‐arm IgM.

**TABLE 3:** Kinetic analysis for the interaction of a group of antibodies with different binding titer with immobilized P‐domain.

Analyte	Immobilization (r.u.)	*K*_on_ (/Ms)	*k*_off_ (/s)	*k*_D_ (M)
1‐arm IgG	138	1.47 × 10^4^	2.50 × 10^−3^	1.70 × 10^−7^
	458	1.02 × 10^4^	2.46 × 10^−3^	2.41 × 10^−7^
	922	8.55 × 10^3^	2.40 × 10^−3^	2.81 × 10^−7^
	1059	1.46 × 10^4^	2.97 × 10^−3^	2.04 × 10^−7^
	3830	2.51 × 10^4^	3.40 × 10^−3^	1.35 × 10^−7^
	4577	1.56 × 10^4^	2.60 × 10^−3^	1.67 × 10^−7^
1‐arm IgM	230	N.D	N.D	N.D
	358	N.D	N.D	N.D
	1534	N.D	N.D	N.D
	3274	N.D	N.D	N.D
2‐arm IgM	245	N.D	N.D	N.D
	687	9.11 × 10^4^	2.63 × 10^−2^	2.89 × 10^−7^
	1652	8.44 × 10^4^	1.22 × 10^−2^	1.44 × 10^−7^
	3372	6.82 × 10^4^	5.56 × 10^−3^	8.15 × 10^−8^
10‐arm IgM	47	2.23 × 10^5^	5.00 × 10^−3^	2.24 × 10^−8^
	624	1.94 × 10^4^	3.55 × 10^−4^	1.83 × 10^−8^
	1427	3.38 × 10^4^	2.19 × 10^−5^	6.49 × 10^−10^
	2149	5.27 × 10^4^	7.19 × 10^−5^	1.36 × 10^−9^
	2520	3.90 × 10^4^	4.74 × 10^−6^	1.22 × 10^−10^
	3384	6.78 × 10^4^	8.88 × 10^−5^	1.31 × 10^−9^

Next, the obtained sensorgrams were examined by comparing the immobilization level of the ligand. SPR analysis revealed a clear valency‐dependent effect on binding. For 1‐arm IgM, affinity was low, and for 1‐arm IgG, affinity remained unchanged across immobilization levels from 50 RU to 4500 RU, confirming that under 1:1 binding conditions, the immobilization density does not influence the affinity. In sharp contrast, 2‐arm IgM and 10‐arm IgM showed dramatic gains—up to 10‐ to 100‐fold—in affinity with increasing immobilization levels. For example, a tenfold rise in immobilization boosted binding strength by 7.2× for 2‐arm IgM and by 25.8× for 10‐arm IgM. Thus, the higher‐valency 10‐arm IgM was 3.6 times more responsive to immobilization density than the 2‐arm form, underscoring the powerful role of multivalency in enhancing antigen engagement (Figure 4b, Table 3). This quantitative demonstration of density‐dependent avidity represents a novel contribution beyond prior qualitative descriptions of multivalency effects in other viral systems (e.g., influenza). Previous studies have not systematically engineered antibodies with identical Fab sequences to isolate valency effects, making this work the first to provide mechanistic insight into how valency and antigen organization synergize to enhance viral recognition.

## 3 CONCLUSION

In this study, we generated and characterized monoclonal IgM and IgG antibodies specific to the capsid proteins of human norovirus strains GII.4 and GII.17. High‐speed AFM directly visualized IgM engaging multiple P‐domain epitopes on intact VLPs, revealing that multivalent binding enables IgM to achieve high functional affinity despite modest intrinsic Fab–antigen affinity. Quantitative SPR analyses using engineered antibodies of defined valency demonstrated that binding affinity for high‐valency IgM increases sharply with antigen density, whereas monovalent IgG binding remains unaffected. While avidity effects of multivalent antibodies are conceptually known, our study uniquely quantifies this phenomenon for norovirus by systematically varying antigen density and antibody valency. This approach reveals that IgM can achieve apparent affinities comparable to high‐affinity IgGs despite modest intrinsic Fab affinity, underscoring the potential of exploiting multivalency in therapeutic antibody design. These findings establish that the dense epitope array on the norovirus capsid strongly amplifies IgM binding through avidity, providing mechanistic insight into how antibody valency and antigen organization synergize to enhance viral recognition. Although future work is needed to define the epitopes of the antibodies, for example through structural approaches, this principle informs the design of vaccines and therapeutics that exploit multivalent engagement for improved potency.

## 4 MATERIALS AND METHODS

### 4.1 Animals

All protocols using mice were approved by the guidelines of the Animal Care and Use Committee, Kyushu University, and performed according to Institutional Guidelines Concerning the Care and Handling of Experimental Animals (protocol code: A23‐372‐1, A25‐232‐0). BALB/c mice (6 weeks old, female) were purchased from SLC (Shizuoka, Japan). All mice were maintained in specific‐pathogen‐free areas (light/dark cycle 12 h/12 h, room temperature 21–23°C, and humidity 50%–60%) and group‐housed (*n* = 2–4 per cage) in a clear plastic cage (15 × 30 × 15 cm) and given free access to food and water.

### 4.2 Preparation of vaccines

After acclimating the mice for 1 week, Norovirus VLP mixture (containing 5 μg each of GII.4 (2012) VLP and GII.17 VLP) was prepared with adjuvant or saline and administered to the mice via intramuscular injection. Two doses were administered at two‐week intervals, and splenocytes were isolated 7 weeks after the start of the experiment.

### 4.3 Preparation of mouse monoclonal antibodies to norovirus VLPs

Splenocytes from mice immunized with norovirus VLPs were isolated and suspended in 10 mL of 10% FBS E‐RDF medium, and filtered through a cell strainer. This was centrifuged (1200 rpm, 5 min), and the pellet fraction was mixed so that the ratio of spleen cells to HAT‐sensitive mouse myeloma cells SP2/0 was 10:1, followed by centrifugation (1200 rpm, 5 min). Resuspend the pellet fraction in 10 mL of 10% FBS E‐RDF medium and centrifuge (1200 rpm, 5 min). Slowly added 1 mL 50% polyethylene glycol (PEG) 1500 to the pellet fraction over 1 min to fuse the cells. Added 10 mL of 10% FBS E‐RDF medium and centrifuged (1200 rpm, 5 min). The pellet fraction was resuspended in 50 mL of 10% FBS E‐RDF medium and seeded in 96‐well plates at 50 μL each. This was incubated in an incubator (37°C, 5% CO_2_) overnight. The next day, 50 μL of HAT medium was added, and the plates were placed in an incubator (37°C, 5% CO_2_) for selection. After 2 weeks, multiple screenings were performed using ELISA on plates showing colonies.

### 4.4 ELISA screening and analysis

Immunoplates (Clear Flat‐Bottom Immuno Nonsterile 96‐Well Plates, Thermo Fisher Scientific) were incubated in coating buffer (15 mM Na_2_CO_3_, 35 mM NaHCO_3_, 3 mM NaN_3_, pH 9.6) (100 μL/well) coated with 1 μg/mL concentrations of a mixture of norovirus VLP GII.4 strain and norovirus VLP GII.17 strain (group 1,2 in primary screening), 1 μg/mL concentration of a mixture of norovirus P‐domain GII.4 strain and norovirus P‐domain GII.17 strain (group 3 in primary screening), 1 μg/mL concentration of norovirus VLP GII.4 strain, norovirus VLP GII.17 strain (group 1,2 in secondary screening, ELISA analysis), 1 μg/mL concentration of norovirus P‐domain GII.4, norovirus P‐domain GII.17 strain (group 3 in secondary screening, ELISA analysis) and 1 μg/mL concentration of ovalbumin (group 1,2,3 in secondary screening, ELISA analysis). Coated plates were sealed and incubated at 4°C overnight, washed three times with 0.05% PBS/Tween‐20 (PBST) solution, and blocked with 300 μL/well of blocking buffer (PBST containing 5% skim milk) for 1 h at 37°C. The plates were then washed three times with PBST and in ELISA screening, incubated with 100 μL of 2‐fold dilution of hybridoma cell culture supernatant in PBST, while in ELISA analysis, incubated with 100 μL of serially diluted concentrations of each anti‐norovirus antibody at 37°C for 1 h. The optimal concentrations of anti‐norovirus antibodies were normalized before testing binding ability and tested at concentrations beginning at 100 μg/mL and then diluted serially. Next, the plates were washed three times with PBST, and horseradish peroxidase‐conjugated goat anti‐mouse IgG (H + L) was added as a secondary antibody and incubated at 37°C for 1 h. Finally, the plate was washed three times with PBST, 60 μL of ABTS chromogenic agent was added, and 30 min later the OD value was measured at 405 nm.

### 4.5 Monoclonalization

The cultured hybridoma cells were measured, and 20,000 cells were resuspended in 2 mL of 10% FBS E‐RDF medium. This was further diluted 20‐fold and 200‐fold, adjusted to 0.5 cells/200 μL, and seeded into a 96‐well plate at 200 μL per well. After incubating for 10 days in an incubator (37°C, 5% CO_2_), ELISA was performed under the same conditions as the previous screening for colonies showing single colonies to confirm success.

### 4.6 Subclass analysis and expression and purification of IgG and IgM mAbs

Hybridoma cell lines producing Abs were gradually expanded from 96‐well plates to 24‐well plates, 6‐well plates, 100 mm dishes, and finally to three 150 mm dishes per cell line. E‐RDF medium at 37°C, 5% CO_2_ for 2 weeks, and the supernatant was used to determine antibody subclasses by Mouse Isotyping Kit (ICLLAB). The monoclonal antibodies produced were subjected to primary purification using HiTrap Protein G HP Columns, HiTrap Protein L Columns, and HiTrap IgM Purification HP Columns. IgG‐type antibodies were purified using HiTrap Protein G HP Columns (Cytiva). The columns were operated at a constant flow rate of 1 mL/min. The column was equilibrated with 10 mL of binding buffer (20 mM sodium phosphate, pH 7.0), the supernatant containing IgG was applied to the column and the column was washed with 10 mL of binding buffer. Elution of bound IgG was performed in a one‐step gradient using 0.1 M glycine buffer, pH 2.7, and 0.5 mL of the eluted fraction was collected. The elution fractions were immediately adjusted to physiological pH by adding 30 μL of 1 M Tris–HCl, pH 9.0. IgM‐type antibodies 12D7 and 3G6 were purified using HiTrap Protein L Columns (Cytiva) and 14A10, 13G1, and 18F9 using HiTrap IgM Purification HP Columns.

For purification using HiTrap Protein L Columns, the column was operated at a constant flow rate of 1 mL/min. The column was equilibrated with 10 mL of binding buffer (20 mM sodium phosphate, 150 mM NaCl, pH 7.2); the supernatant containing IgM was applied to the column and the column was washed with 10 mL of binding buffer. Elution of bound IgM was performed in a one‐step gradient with 0.1 M sodium citrate, pH 2.7 and 0.5 mL of the eluted fraction was collected. The elution fraction was immediately adjusted to physiological pH by adding 70 μL of 1 M Tris–HCl, pH 9.0. For purification using the HiTrap IgM Purification HP Column, ammonium sulfate was first added to the supernatant containing IgM to a final concentration of 0.8 M. To avoid precipitation of IgM, the sample was supplemented with a small amount of individual ammonium sulfate while continuously expanding the sample. The sample was passed through a 0.80 μm filter. The column was operated at a constant flow rate of 1 mL/min. The column was equilibrated with 10 mL of binding buffer (20 mM sodium phosphate, 0.8 M ammonium sulfate, pH 7.5). supernatant containing IgM was applied to the column and the column was washed with 10 mL of binding buffer. Elution of bound IgG was performed in a one‐step gradient with 20 mM sodium phosphate, pH 7.5, and 0.5 mL of the eluted fraction was collected. Antibodies 12D7, 14A10, 13G1 14C2, 18F9, and 5H8 were purified using HiLoad 16/600 Superdex 200 pg and 3G6 was purified using HiLoad 16/600 Superdex 75 pg. after primary purification by column chromatography.

### 4.7 High‐speed atomic force microscopy (HS‐AFM)

HS‐AFM was conducted in tapping mode at room temperature using a micro‐cantilever (BL‐AC7DS, Olympus Corporation, Tokyo, Japan) with a resonance frequency of approximately 600 kHz, a spring constant of approximately 0.2 N/m, and a Q factor of approximately 1.0. To investigate the interaction between norovirus GII.4 VLPs and antibody 14A10, norovirus GII.4 VLPs were immobilized on mica treated with 3‐aminopropyltriethoxysilane (APTES). A freshly cleaved mica surface was first treated with a 2 μL droplet of 1% APTES and incubated for 3 min. Following a wash with MilliQ water, the APTES‐treated substrate was incubated with norovirus GII.4 VLPs for 5 min. Unbound VLPs were subsequently washed away with PBS. HS‐AFM imaging was then performed in the same buffer, during which antibody 14A10 was added to observe its interaction with the immobilized VLPs. Images were recorded at a frame rate of 0.12 s per frame.

To generate the pseudo‐AFM image shown in Figure 1b, a collision simulation was performed between the structural model and a virtual AFM tip using pyNuD, a Python‐based viewer software developed in our laboratory. The tip was modeled as a conical shape with a radius of curvature of 1 nm and a cone angle of 10°. The resulting simulated image was subsequently processed with a low‐pass filter with a spatial frequency of 5 nm^−1^ to approximate the experimental resolution. In constructing the structural model of IgM, the pentameric Fc region was based on PDB ID: 8BPF. For template structures other than the pentameric Fc region of IgM, we used PDB ID: 7XQ8. The amino acid sequences of the Fab regions were replaced with the heavy (H‐chain) and light (L‐chain) chain sequences of the 14A10 IgM, and homology modeling was performed using SWISS‐MODEL (SWISS‐MODEL Interactive Workspace). Using the constructed IgM structural model together with the norovirus capsid structure (PDB ID: 7MRY), we generated a complex model of IgM bound to the norovirus capsid.

For image processing and quantitative analysis of the movement of IgM on the VLP surface, all HS‐AFM data were processed using pyNuD. The centroid of individual IgM molecules was tracked across successive frames using a custom‐made program to obtain two‐dimensional trajectories. From these trajectories (*n* = 28), the ensemble‐averaged MSD was calculated. The initial diffusion coefficient was determined by a linear fit to the MSD curve at short time lags (0.12–0.60 s) using the equation MSD(Δτ) = 4D Δτ. To estimate the exploration area of IgM on the VLP, the plateau value of the MSD curve (MSD_max_) was used to calculate the radius of the confinement area (*a*) based on the relationship MSD_max_ = *a* ^2^/2. Furthermore, binding dwell times were measured for 31 independent events to evaluate the binding stability, with events exceeding the 90‐s observation window treated as censored data.

### 4.8 Expression and purification of norovirus P‐domains

To determine the specificity of anti‐norovirus binding antibodies, DNA fragments encoding the P‐domain (amino acid residues 224‐530) of the VP1 protein from norovirus GII.4 and GII.17 were cloned into a modified pMAL‐c6T (New England Biolabs) vector. The amino acid sequences for these P‐domains, corresponding to GenBank accession numbers AQT25659 and AKI30060, respectively, are provided in Supplementary Data S1. A His‐tag sequence was engineered directly at the N‐terminus of the P‐domain, utilizing the pMAL‐c6T vector backbone. For protein expression, the resulting plasmids were transformed into competent *Escherichia coli* BL21 (DE3) cells. Expression was induced with isopropyl‐*β*‐D‐thiogalactopyranoside (IPTG) at a final concentration of 0.3 mM for 18 h at 16°C. The His‐tagged fusion‐P‐domain protein was purified using a Ni‐NTA Agarose (FUJIFILM). The P‐domain was further purified by SEC with a HiLoad 16/600 Superdex 75 pg column.

### 4.9 Expression and purification of 1‐arm IgM, 2‐arm IgM and 1‐arm IgG

The sequences of the Fab region of IgM antibody 14A10, the sequence of the Fab region of 14A10 with the Fc region sequence of trastuzumab added, and the sequence of the Fab region of IgG antibody 14C2 were recombinantly expressed as 1‐arm IgM, 2‐arm IgM, and 1‐arm IgG, respectively. The Fab region of the heavy chain of IgM antibody 14A10 consisted of the variable domain of the heavy chain and the first constant domain (C*μ*1). The variable domain sequence contained 122 amino acid residues, corresponding to residues 1–114 based on Kabat numbering (Wu & Kabat, 1970). The Kabat numbering was performed using the online tool AbRSA (Li et al., 2019). The C*μ*1 domain sequence, which was used for this study, was identified to be 104 amino acid residues in length and showed 100% identity with the full‐length IgM constant region sequence (GenBank accession number, P01872). The Fc region of trastuzumab used for this study corresponds to amino acid residues 224‐530 of the human IgG1 heavy chain, based on GenBank accession number WDS83891. The amino acid sequences of 1‐arm IgM, 2‐arm IgM, and 1‐arm IgG are provided in Supplementary Data S2. The gene sequence for 1‐arm IgG was codon‐optimized for expression in mammalian cells. The sequences for the 1‐arm and 2‐arm IgM were not codon‐optimized.

These antibodies were expressed in Expi293 cells and purified using Ni‐NTA Agarose (FUJIFILM) for 1‐arm IgM and 1‐arm IgG, or Protein A (Cytiva) for 2‐arm IgM. The recombinant proteins were further purified by SEC with a HiLoad 16/600 Superdex 75 pg. column.

### 4.10 Binding analysis by SPR

SPR analysis was performed using a Biacore 8K system (GE Healthcare). Purified norovirus P‐domain from GII.4 and GII.17 strains were immobilized to a CM5 sensor chip using the amine coupling method. PBST was used as reaction buffer, and the flow rate was 30 μL/min. The affinity constant (*K*_*D*_) is equal to the ratio of the rate constants (*K*_*D*_ = *k*_*d*_/*k*_*a*_, association rate constant (*k*_*a*_), dissociation rate constant (*k*_*d*_)) and the antigen–antibody binds in a 1:1 kinetic model. To measure binding to norovirus P‐domain GII.4 and GII.17 strains, the anti‐norovirus binding antibody produced was serially diluted 3‐fold to 1.23 nM or 2‐fold to 25 nM in PBST at a rate of 30 μL/min for 180 s. Dissociation was allowed to proceed for 600 s. The chip surface was regenerated with MgCl_2_ at a concentration of 3 M. The binding curves were fitted as a 1:1 kinetics mode by Biacore Insight Evaluation Software (Cytiva, version 3.0.12.15655).

## AUTHOR CONTRIBUTIONS

**Jumpei Tagawa:** Conceptualization; writing – original draft; investigation; data curation; formal analysis. **Saeko Yanaka:** Conceptualization; writing – original draft; formal analysis; investigation; funding acquisition; project administration; supervision. **Yuri Kato:** Writing – review and editing; investigation. **Akitsu Masuda:** Writing – review and editing; investigation. **Jae Man Lee:** Writing – review and editing; investigation. **Akinobu Senoo:** Writing – review and editing; investigation. **Kosuke Oyama:** Writing – review and editing; investigation. **Takayuki Uchihashi:** Writing – review and editing; investigation; methodology; formal analysis; data curation. **Motohiro Nishida:** Writing – review and editing; resources; project administration; methodology; investigation. **Takahiro Kusakabe:** Writing – review and editing; funding acquisition; project administration; resources; investigation; methodology. **Jose M. M. Caaveiro:** Conceptualization; writing – original draft; investigation; funding acquisition; formal analysis; supervision; project administration.

## FUNDING INFORMATION

This work was supported in part by the Strategic Center of Biomedical Advanced Vaccine Research and Development for Preparedness and Response from the Japan Agency for Medical Research and Development AMED (SCARDA JP233fa827004 to T.U., M.N., T.K., and J.M.M.C., JP21ae0121020 and JP23ak0101209 to S.Y.), MEXT/JSPS Grants‐in‐Aid for Scientific Research (JP22H02755 and JP25H02252 to S.Y. and JP20H03228 to J.M.M.C), MEXT Promotion of Development of a Joint Usage/Research System Project: Coalition of Universities for Research Excellence Program (CURE) Grant Number JPMXP1323015482, the Joint Research of the Exploratory Research Center on Life and Living Systems (ExCELLS) (ExCELLS programs 23EXC312 and 24EXC341 to J.M.M.C.), the Platform Project for Supporting Drug Discovery and Life Science Research [Basis for Supporting Innovative Drug Discovery and Life Science Research] (BINDS) from AMED (JP23ama121031 to J.M.M.C.), the MEXT Design and Engineering by Joint Inverse Innovation for Materials Architecture project, and JST SPRING, Japan Grant Number JPMJSP2180.

## CONFLICT OF INTEREST STATEMENT

The authors declare no competing interests.

## Supporting information


**Supplementary Movie 1** HS‐AFM movie of the interaction of 14A10 IgM with VLPs of norovirus GII.4 strains.
This movie shows the dynamic binding and lateral movement of a single 14A10 IgM molecule on the surface of a norovirus GII.4 VLP, corresponding to the snapshots shown in Figure 1a. The video captures a complete sequence of events: the initial binding of the IgM to the VLP surface, its subsequent lateral movement (scanning), and its eventual dissociation from the capsid. The frame at 4.2 s was used for the high‐magnification analysis in Figure 1b. Image speed: 0.12 s/frame.

**Supplementary Movie 2** Centroid tracking of the IgM molecule during surface exploration.
This movie provides the tracking analysis for the IgM molecule shown in Supplementary Movie 1. The red marker indicates the tracked centroid position of the antibody as it moves across the VLP surface. These coordinates were utilized for the ensemble‐averaged mean square displacement (MSD) analysis presented in Figure 1c to characterize the confined diffusion behavior. The tracking begins from the binding event and continues until just before dissociation.

**Data S1:** Supporting Information.

## Data Availability

The data that supports the findings of this study are available in the supplementary material of this article.

## References

[pro70522-bib-0001] Alvarado G , Ettayebi K , Atmar RL , Bombardi RG , Kose N , Estes MK , et al. Human monoclonal antibodies that neutralize pandemic GII.4 noroviruses. Gastroenterology. 2018;155:1898–1907. 30170116 10.1053/j.gastro.2018.08.039 PMC6402321

[pro70522-bib-0002] Alvarado G , Salmen W , Ettayebi K , Hu L , Sankaran B , Estes MK , et al. Broadly cross‐reactive human antibodies that inhibit genogroup I and II noroviruses. Nat Commun. 2021;12:4320. 34262046 10.1038/s41467-021-24649-w PMC8280134

[pro70522-bib-0003] Bányai K , Estes MK , Martella V , Parashar UD . Viral gastroenteritis. Lancet. 2018;392:175–186. 30025810 10.1016/S0140-6736(18)31128-0 PMC8883799

[pro70522-bib-0004] Bartsch SM , Lopman BA , Ozawa S , Hall AJ , Lee BY . Global economic burden of norovirus gastroenteritis. PLoS One. 2016;11:e0151219. 27115736 10.1371/journal.pone.0151219 PMC4846012

[pro70522-bib-0005] Cannon JL , Bonifacio J , Bucardo F , Buesa J , Bruggink L , Chan MC‐W , et al. Global trends in norovirus genotype distribution among children with acute gastroenteritis. Emerg Infect Dis. 2021;27:1438–1445. 33900173 10.3201/eid2705.204756 PMC8084493

[pro70522-bib-0006] Chan MCW , Lee N , Hung T‐N , Kwok K , Cheung K , Tin EKY , et al. Rapid emergence and predominance of a broadly recognizing and fast‐evolving norovirus GII.17 variant in late 2014. Nat Commun. 2015;6:10061. 26625712 10.1038/ncomms10061 PMC4686777

[pro70522-bib-0007] Chhabra P , de Graaf M , Parra GI , Chan MC‐W , Green K , Martella V , et al. Updated classification of norovirus genogroups and genotypes. J Gen Virol. 2019;100:1393–1406. 31483239 10.1099/jgv.0.001318 PMC7011714

[pro70522-bib-0008] Czakó, R , Atmar, RL , Opekun, AR , Gilger, MA , Graham, DY , Estes, MK (2015) Experimental human infection with Norwalk virus elicits a surrogate neutralizing antibody response with cross‐genogroup activity. Clin Vaccine Immunol 22:221–228. 25540269 10.1128/CVI.00516-14 PMC4308873

[pro70522-bib-0009] Dai Y‐C , Xia M , Huang Q , Tan M , Qin L , Zhuang Y‐L , et al. Characterization of antigenic relatedness between GII.4 and GII.17 noroviruses by use of serum samples from norovirus‐infected patients. J Clin Microbiol. 2017;55:3366–3373. 28904188 10.1128/JCM.00865-17 PMC5703803

[pro70522-bib-0010] De Graaf M , Van Beek J , Vennema H , Podkolzin AT , Hewitt J , Bucardo F , et al. Emergence of a novel GII.17 norovirus—end of the GII.4 era? Euro Surveill. 2015;20:21178. 10.2807/1560-7917.ES2015.20.26.21178 26159308 PMC5921880

[pro70522-bib-0011] Desai R , Hembree CD , Handel A , Matthews JE , Dickey BW , McDonald S , et al. Severe outcomes are associated with genogroup 2 genotype 4 norovirus outbreaks: a systematic literature review. Clin Infect Dis. 2012;55:189–193. 22491335 10.1093/cid/cis372 PMC3491774

[pro70522-bib-0012] Du J , Gu Q , Liu Y , Li Q , Guo T , Liu Y . The endemic GII.4 norovirus‐like‐particle induced‐antibody lacks of cross‐reactivity against the epidemic GII.17 strain. J Med Virol. 2021;93:3974–3979. 32869863 10.1002/jmv.26474 PMC8246737

[pro70522-bib-0013] Gray JJ , Cunliffe C , Ball J , Graham DY , Desselberger U , Estes MK . Detection of immunoglobulin M (IgM), IgA, and IgG Norwalk virus‐specific antibodies by indirect enzyme‐linked immunosorbent assay with baculovirus‐expressed Norwalk virus capsid antigen in adult volunteers challenged with Norwalk virus. J Clin Microbiol. 1994;32:3059–3063. 7883902 10.1128/jcm.32.12.3059-3063.1994 PMC264229

[pro70522-bib-0014] Keyt BA , Baliga R , Sinclair AM , Carroll SF , Peterson MS . Structure, function, and therapeutic use of IgM antibodies. Antibodies. 2020;9:53. 33066119 10.3390/antib9040053 PMC7709107

[pro70522-bib-0015] Li L , Chen S , Miao Z , Liu Y , Liu X , Xiao Z , et al. AbRSA: a robust tool for antibody numbering. Protein Sci. 2019;28:1524–1531. 31020723 10.1002/pro.3633 PMC6635766

[pro70522-bib-0016] Lindesmith L , Moe C , LePendu J , Frelinger JA , Treanor J , Baric RS . Cellular and humoral immunity following Snow Mountain virus challenge. J Virol. 2005;79:2900–2909. 15709009 10.1128/JVI.79.5.2900-2909.2005 PMC548455

[pro70522-bib-0017] Lindesmith LC , Beltramello M , Donaldson EF , Corti D , Swanstrom J , Debbink K , et al. Immunogenetic mechanisms driving norovirus GII.4 antigenic variation. PLoS Pathog. 2012;8:e1002705. 22615565 10.1371/journal.ppat.1002705 PMC3355092

[pro70522-bib-0018] Lindesmith LC , Donaldson EF , LoBue AD , Cannon JL , Zheng D‐P , Vinje J , et al. Mechanisms of GII.4 norovirus persistence in human populations. PLoS Med. 2008;5:e31. 18271619 10.1371/journal.pmed.0050031 PMC2235898

[pro70522-bib-0019] Lindesmith LC , McDaniel JR , Changela A , Verardi R , Kerr SA , Costantini V , et al. Sera antibody repertoire analyses reveal mechanisms of broad and pandemic strain neutralizing responses after human norovirus vaccination. Immunity. 2019;50:1530–1541.e8. 31216462 10.1016/j.immuni.2019.05.007 PMC6591005

[pro70522-bib-0020] Liu T , Hsiung J , Zhao S , Kost J , Sreedhar D , Hanson CV , et al. Quantification of antibody avidities and accurate detection of SARS‐CoV‐2 antibodies in serum and saliva on plasmonic substrates. Nat Biomed Eng. 2020;4:1188–1196. 33122853 10.1038/s41551-020-00642-4

[pro70522-bib-0021] Oostindie SC , Lazar GA , Schuurman J , Parren PWHI . Avidity in antibody effector functions and biotherapeutic drug design. Nat Rev Drug Discov. 2022;21:715–735. 35790857 10.1038/s41573-022-00501-8 PMC9255845

[pro70522-bib-0022] Paloni M , Cavallotti C . Molecular modeling of the interaction of protein L with antibodies. ACS Omega. 2017;2:6464–6472. 31457247 10.1021/acsomega.7b01123 PMC6645367

[pro70522-bib-0023] Parra GI , Azure J , Fischer R , Bok K , Sandoval‐Jaime C , Sosnovtsev SV , et al. Identification of a broadly cross‐reactive epitope in the inner Shell of the norovirus capsid. PLoS One. 2013;8:e67592. 23805319 10.1371/journal.pone.0067592 PMC3689733

[pro70522-bib-0024] Patel MM , Widdowson M‐A , Glass RI , Akazawa K , Vinjé J , Parashar UD . Systematic literature review of role of noroviruses in sporadic gastroenteritis. Emerg Infect Dis. 2008;14:1224–1231. 18680645 10.3201/eid1408.071114 PMC2600393

[pro70522-bib-0025] Pletneva MA , Sosnovtsev SV , Green KY . The genome of Hawaii virus and its relationship with other members of the caliciviridae. Virus Genes. 2001;23:5–16. 11556401 10.1023/a:1011138125317

[pro70522-bib-0026] Prasad BVV , Hardy ME , Dokland T , Bella J , Rossmann MG , Estes MK . X‐ray crystallographic structure of the Norwalk virus capsid. Science. 1999;286:287–290. 10514371 10.1126/science.286.5438.287

[pro70522-bib-0027] Robilotti E , Deresinski S , Pinsky BA . Norovirus. Clin Microbiol Rev. 2015;28:134–164. 25567225 10.1128/CMR.00075-14 PMC4284304

[pro70522-bib-0028] Sapparapu G , Czakó R , Alvarado G , Shanker S , Prasad BVV , Atmar RL , et al. Frequent use of the IgA isotype in human B cells encoding potent norovirus‐specific monoclonal antibodies that block HBGA binding. PLoS Pathog. 2016;12:e1005719. 27355511 10.1371/journal.ppat.1005719 PMC4927092

[pro70522-bib-0029] Strother CA , Brewer‐Jensen PD , Becker‐Dreps S , Zepeda O , May S , Gonzalez F , et al. Infant antibody and B‐cell responses following confirmed pediatric GII.17 norovirus infections functionally distinguish GII.17 genetic clusters. Front Immunol. 2023;14:1229724. 37662930 10.3389/fimmu.2023.1229724 PMC10471973

[pro70522-bib-0030] Tan M , Jiang X . Norovirus and its histo‐blood group antigen receptors: an answer to a historical puzzle. Trends Microbiol. 2005;13:285–293. 15936661 10.1016/j.tim.2005.04.004

[pro70522-bib-0031] Tanaka T , Kitamoto N , Jiang X , Estes MK . High efficiency cross‐reactive monoclonal antibody production by Oral immunization with recombinant Norwalk virus‐like particles. Microbiol Immunol. 2006;50:883–888. 17116984 10.1111/j.1348-0421.2006.tb03864.x

[pro70522-bib-0032] van Loben Sels JM , Green KY . The antigenic topology of norovirus as defined by B and T cell epitope mapping: Implications for universal vaccines and therapeutics. Viruses. 2019;11:432. 31083353 10.3390/v11050432 PMC6563215

[pro70522-bib-0033] Vongpunsawad S , Venkataram Prasad BV , Estes MK . Norwalk virus minor capsid protein VP2 associates within the VP1 Shell domain. J Virol. 2013;87:4818–4825. 23408637 10.1128/JVI.03508-12 PMC3624303

[pro70522-bib-0034] Wibroe PP , Helvig SY , Moein Moghimi S . The role of complement in antibody therapy for infectious diseases. Microbiol Spectr. 2014;2(2):10.1128. 10.1128/microbiolspec.AID-0015-2014 26105816

[pro70522-bib-0035] Wu TT , Kabat EA . An analysis of the sequences of the variable regions of BENCE jones proteins and myeloma light chains and their IMPLICATIONS for antibody complementarity. J Exp Med. 1970;132:211–250. 5508247 10.1084/jem.132.2.211 PMC2138737

[pro70522-bib-0036] Yi Y , Wang X , Wang S , Xiong P , Liu Q , Zhang C , et al. Identification of a blockade epitope of human norovirus GII.17. Emerg Microbes Infect. 2021;10:954–963. 33929932 10.1080/22221751.2021.1925162 PMC8143627

