# Supplementary material for: Characterization of high affinity IgM and IgG monoclonal antibodies against norovirus variants GII.4 and GII.17
Source: Protein Sci. 2026 Mar 12;35(4):e70522. doi: 10.1002/pro.70522 (PMC13140594; doi:10.1002/pro.70522)
Supplement: Supplementary file 3 — Data S1: Supporting Information. [file PRO-35-e70522-s002.docx]

**Characterization of high affinity IgM and IgG monoclonal antibodies against norovirus variants GII.4 and GII.17.**

Jumpei Tagawa^1,2^, Saeko Yanaka^*,1,2^, Yuri Kato^1^, Akitsu Masuda^3^, Jae Man Lee^3^, Akinobu Senoo^1^, Kosuke Oyama^4^, Takayuki Uchihashi^5^, Motohiro Nishida^1^, Takahiro Kusakabe^6^, Jose M.M. Caaveiro^*,1^

*^1^Graduate School of Pharmaceutical Sciences, Kyushu University, 3-1-1 Maidashi, Higashi, Fukuoka, 812-8582, Japan,* *^2^Laboratory for Materials and Structures,* *Institute of Integrated research, Institute of Science Tokyo, 4259 Nagatsuda, Midori-ku, Yokoyama, Kanagawa, 226-8503, Japan, ^3^Laboratory of Creative Science for Insect Industries, Kyushu University Graduate School of Bioresource and Bioenvironmental Sciences, 744 Motooka, Nishi-ku, Fukuoka, 819-0395, Japan, ^4^Department of Biological Science and Technology, Tokyo University of Science, 6-3-1 Shinjuku, Katsushika-ku, Tokyo, 125-8585, Japan,^5^Graduate School of Sciences, Nagoya University, Furocho, Chikusa, Nagoya, 464-8602 Aichi, Japan, ^6^Laboratory of Insect Genome Science, Kyushu University Graduate School of Bioresource and Bioenvironmental Sciences, 744 Motooka, Nishi-ku, Fukuoka, 819-0395, Japan,*

^*^Corresponding author: Jose M.M. Caaveiro ([jose@phar.kyushu-u.ac.jp](mailto:jose@phar.kyushu-u.ac.jp)) and Saeko Yanaka (yanaka.s.ab@m.titech.ac.jp)

**
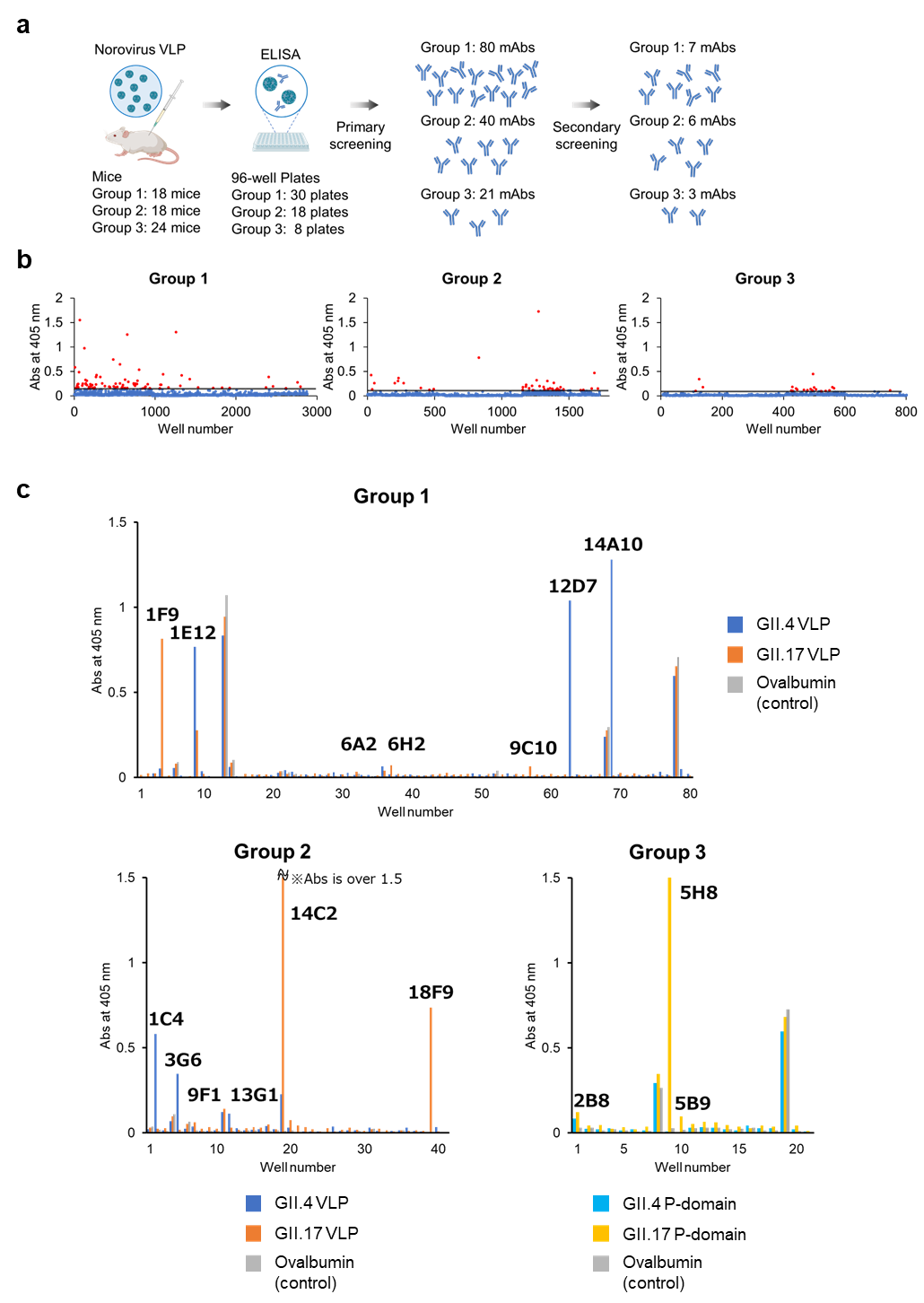
**

**Supplementary Figure 1 Selection of hybridoma cells producing anti-norovirus antibodies.**

(a) Mice immunized with norovirus VLPs were sacrificed and plasma cells from the spleens of immunized mice were fused with myeloma cells to create hybridoma cells expressing mAbs. Cells producing Abs that reacted with wells decorated with norovirus protein were identified by ELISA. A total of 141 wells identified in the primary screening. Secondary screening also employing ELISA identified 16 mAbs, which excluded Abs with unspecific binding and false positives from the primary screening. Samples belonging to groups 1 and 2 were evaluated against norovirus VLPs, whereas samples belonging to group 3 were selected against the P-domain. (b) ELISA corresponding to the primary screening; red indicates clones that were used for further analysis. (c) ELISA for secondary screening, with the names of clones used for further analysis.


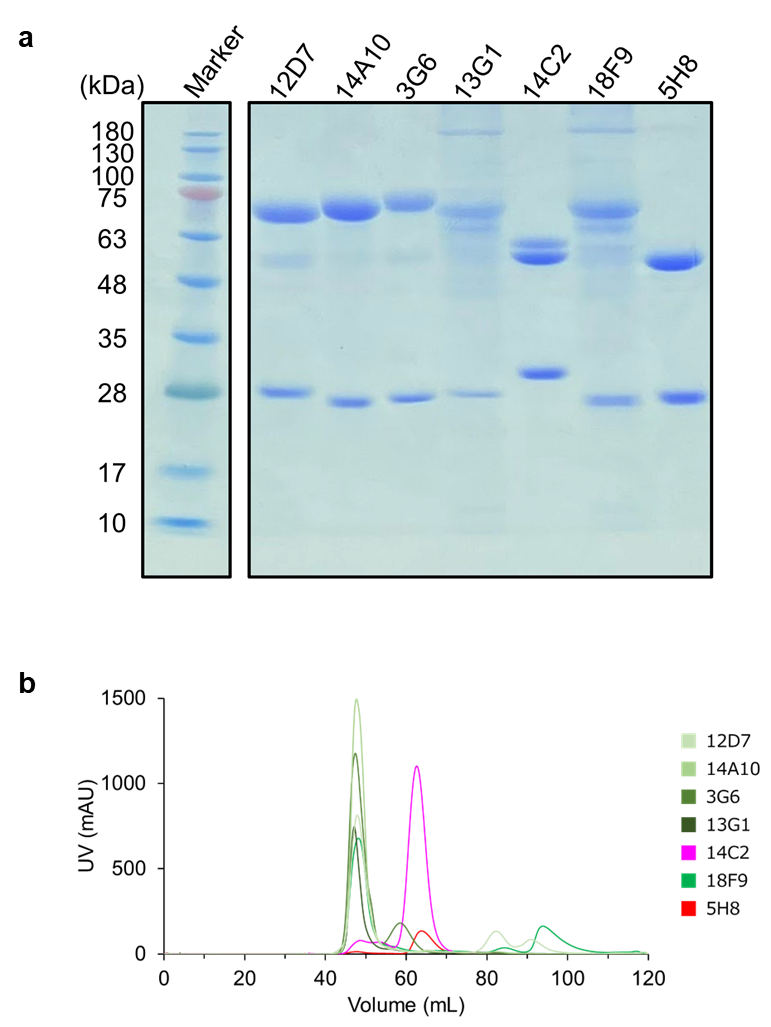


**Supplementary Figure 2 Purification of anti-norovirus monoclonal antibodies.**

(a) Overlaid chromatograms from size exclusion chromatography of mAbs, with IgM-type antibodies shown in green and IgG-type antibodies in red. (b) Reduced SDS-PAGE analysis of purified mAbs.


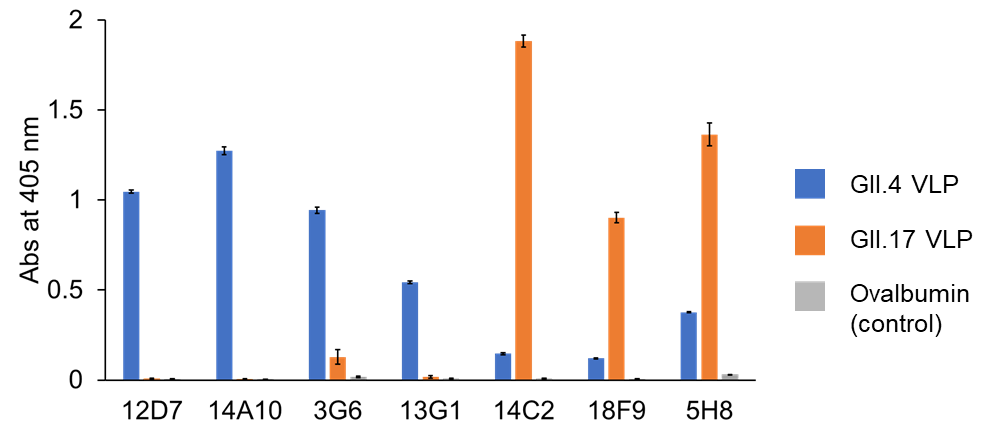


**Supplementary Figure 3 Evaluation of binding specificity of anti-norovirus antibodies by ELISA.**

Indirect ELISA was used to evaluate the binding of seven mouse mAbs to GII.4 VLP and GII.17 VLP, respectively. Error bars indicate standard deviation. (n=3)


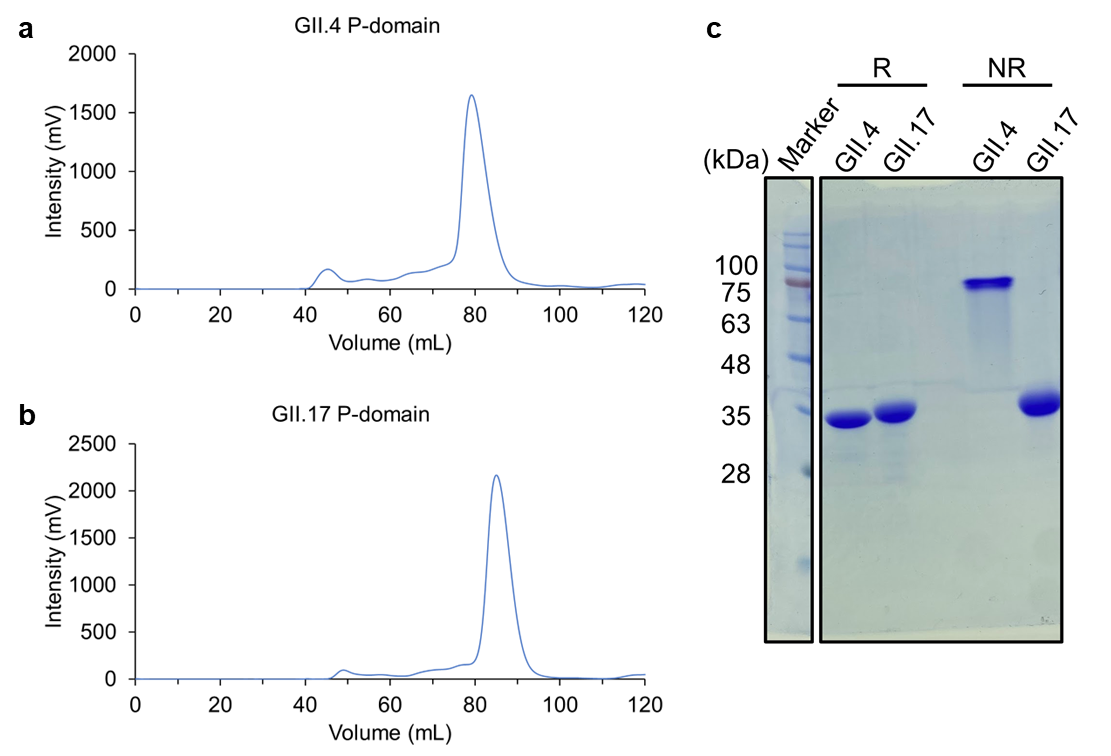


**Supplementary Figure 4 Purification of norovirus P-domain.**

(a) Chromatogram from size exclusion chromatography of P-domain of norovirus GII.4 strain. (b) Chromatogram from size exclusion chromatography of P-domain of norovirus GII.17 strain. (c) SDS-PAGE analysis of purified norovirus P-domain.


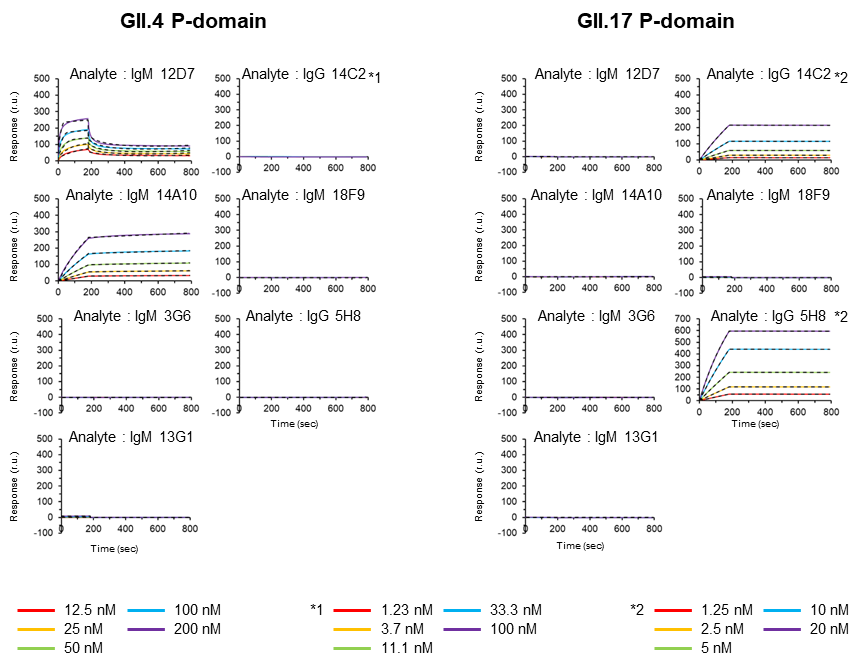


**Supplementary Figure 5 Binding of anti-norovirus monoclonal antibodies to the capsid P-domain.**

Binding of mAbs to norovirus P-domain was evaluated by SPR. Sensorgrams of binding to GII.4 strains are shown on the left and those to GII.17 strains on the right. The solid line shows the obtained sensorgrams and the fitting is shown as a dashed line.


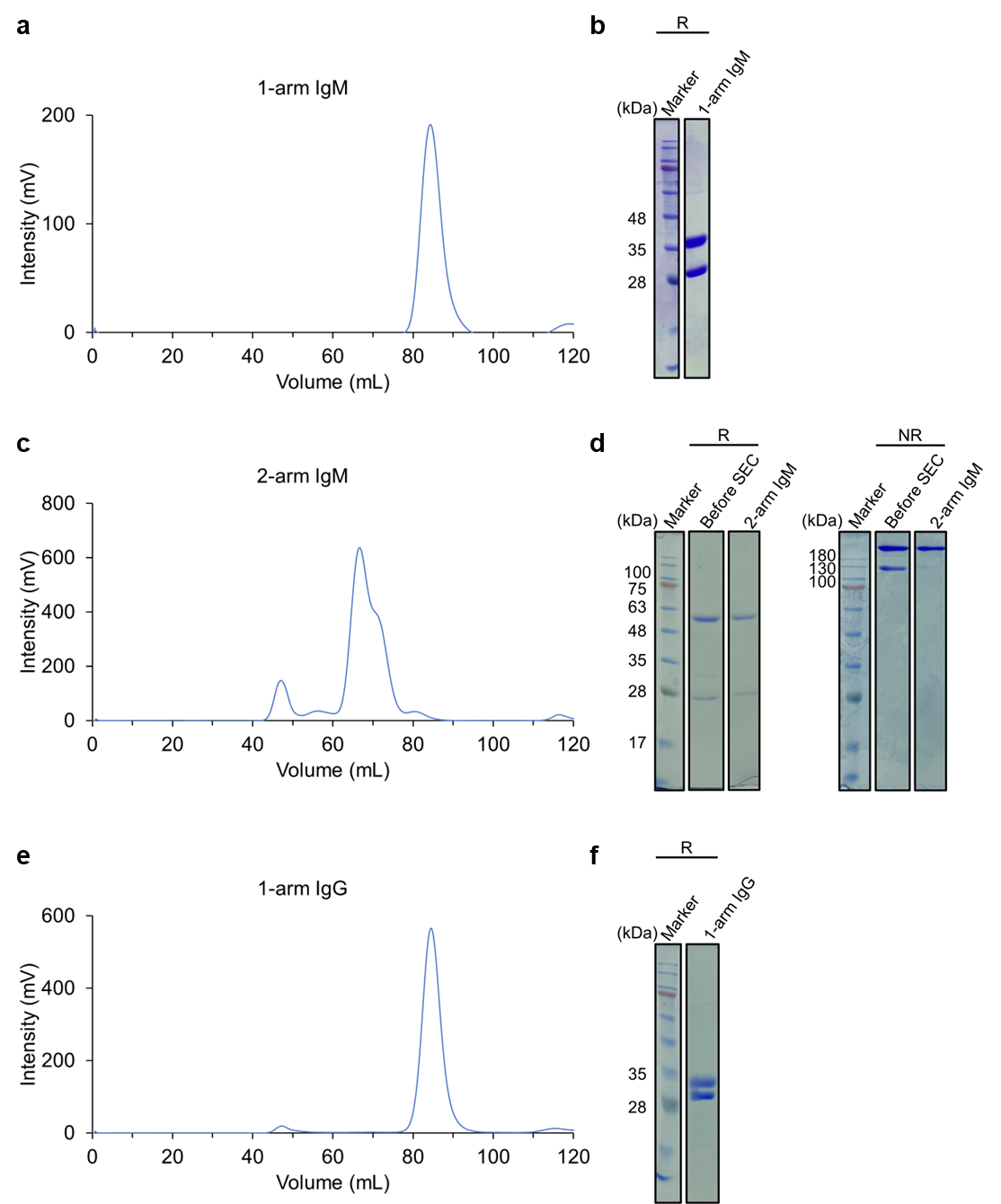


**Supplementary Figure 6 Purification of 1-arm IgM, 2-arm IgM and 1-arm IgG.**

(a) Chromatogram from size exclusion chromatography of 1-arm IgM. (b) SDS-PAGE analysis of purified 1-arm IgM. (c) Chromatogram from size exclusion chromatography of 2-arm IgM. (d) SDS-PAGE analysis of purified 2-arm IgM. (e) Chromatogram from size exclusion chromatography of 1-arm IgG. (f) SDS-PAGE analysis of purified 1-arm IgG.

**Supplementary Movie 1 | HS-AFM movie of the interaction of 14A10 IgM with VLPs of norovirus GII.4 strains.**

This movie shows the dynamic binding and lateral movement of a single 14A10 IgM molecule on the surface of a norovirus GII.4 VLP, corresponding to the snapshots shown in Figure 1a. The video captures a complete sequence of events: the initial binding of the IgM to the VLP surface, its subsequent lateral movement (scanning), and its eventual dissociation from the capsid. The frame at 4.2 s was used for the high-magnification analysis in Figure 1b. Image speed: 0.12 s/frame.

**Supplementary Movie 2 | Centroid tracking of the IgM molecule during surface exploration.**

This movie provides the tracking analysis for the IgM molecule shown in Supplementary Movie 1. The red marker indicates the tracked centroid position of the antibody as it moves across the VLP surface. These coordinates were utilized for the ensemble-averaged mean square displacement (MSD) analysis presented in Figure 1c to characterize the confined diffusion behavior. The tracking begins from the binding event and continues until just before dissociation.

**Supplementary Data S1. Amino acid sequences of Norovirus P-domain.**

The following are the amino acid sequences of the P-domains from norovirus GII.4 and GII.17 used in this study.

1. Norovirus GII.4 P-domain

MKIHHHHHHTKPFSVPVLTVEEMTNSRFPIPLEKLFTGPSSAFVVQPQNGRCTTDGVLLGTTQLSPVNICTFRGDVTHITGSHNYTMNLASQNWSNYDPTEEIPAPLGTPDFVGKIQGMLTQTTRTDGSTRGHKATVYTGSADFAPKLGRVQFETDTNNDFEANQNTKFTPVGVIQDGGTTHRNEPQQWVLPSYSGRNTPNVHLAPAVAPTFPGEQLLFFRSTMPGCSGYPNMDLDCLLPQEWVQYFYQEAAPAQSDVALLRFVNPDTGRVLFECKLHKSGYVTVAHTGQHDLVIPPNGYFRFDSWVNQFYTLAPM

1. Norovirus GII.17 P-domain

MKIHHHHHHTKPFSLPILTLSELTNSRFPVPIDSLFTAQNNVLQVQCQNGRCTLDGELQGTTQLLPSGICAFRGRVTAQINQRDRWHMQLQNLNGTTYDPTDDVPAPLGTPDFKGVVFGMVSQRNVGNDAPGSTRAQQAWVSTYSPQFVPKLGSINLRISDNDDFQFQPTKFTPVGVNDDDDGHPFRQWELPNYSGELTLNMNLAPPVAPNFPGEQLLFFRSFVPCSGGYNQGIIDCLIPQEWIQHFYQESAPSQSDVALIRYVNPDTGRTLFEAKLHRSGYITVAHSGDYPLVVPANGHFRFDSWVNQFYSLAPM

**Supplementary Data S2. Amino acid sequences of 1-arm IgM, 2-arm IgM and 1-arm IgG.**

The following are the amino acid sequences of 1-arm IgM, 2-arm IgM and 1-arm IgG used in this study.

1. Heavy chain of 1-arm IgM

EVQLQQSGPELVKPGASMKISCKASGYSFTGYTMNWVKQSHGKNLEWIGLINPYNGGTSYNQKFKGKATLTVDKSSSTAYMELLSLTSEDSAVYYCARGGVVADYYAMDYWGQGTSVTVSSESQSFPNVFPLVSCESPLSDKNLVAMGCLARDFLPSTISFTWNYQNNTEVIQGIRTFPTLRTGGKYLATSQVLLSPKSILEGSDEYLVCKIHYGGKNRDLHVPIPHHHHHH

1. Light chain of 1-arm IgM DIVLTQSPAIMSASPGEKVTMTCSASSSVSYMHWYQQKSGTSPKRWIYDTSKLASGVPARFSGSGSGTSYSLTISSMEAEDAATYYCQQWSSNPLTFGAGTKLELKRADAAPTVSIFPPSSEQLTSGGASVVCFLNNFYPKDINVKWKIDGSERQNGVLNSWTDQDSKDSTYSMSSTLTLTKDEYERHNSYTCEATHKTSTSPIVKSFNRNE
2. Heavy chain of 2-arm IgM EVQLQQSGPELVKPGASMKISCKASGYSFTGYTMNWVKQSHGKNLEWIGLINPYNGGTSYNQKFKGKATLTVDKSSSTAYMELLSLTSEDSAVYYCARGGVVADYYAMDYWGQGTSVTVSSESQSFPNVFPLVSCESPLSDKNLVAMGCLARDFLPSTISFTWNYQNNTEVIQGIRTFPTLRTGGKYLATSQVLLSPKSILEGSDEYLVCKIHYGGKNRDLHVPIPDKTHTCPPCPAPELLGGPSVFLFPPKPKDTLMISRTPEVTCVVVDVSHEDPEVKFNWYVDGVEVHNAKTKPREEQYNSTYRVVSVLTVLHQDWLNGKEYKCKVSNKALPAPIEKTISKAKGQPREPQVYTLPPSRDELTKNQVSLTCLVKGFYPSDIAVEWESNGQPENNYKTTPPVLDSDGSFFLYSKLTVDKSRWQQGNVFSCSVMHEALHNHYTQKSLSLSPGK
3. Light chain of 2-arm IgM

DIVLTQSPAIMSASPGEKVTMTCSASSSVSYMHWYQQKSGTSPKRWIYDTSKLASGVPARFSGSGSGTSYSLTISSMEAEDAATYYCQQWSSNPLTFGAGTKLELKRADAAPTVSIFPPSSEQLTSGGASVVCFLNNFYPKDINVKWKIDGSERQNGVLNSWTDQDSKDSTYSMSSTLTLTKDEYERHNSYTCEATHKTSTSPIVKSFNRNEC

1. Heavy chain of 1-arm IgG

QAYLQQSGAELMKPGASMRISCKATGYTFSNYWIEWVKQRPGHGLEWIGEILPGGGGTDYNEKFKGKATFTADTSSNTAYMQLSSLTSEDSAVYYCARSTFYGYDGGLYYFPMDYWGQGTSVTVSPAKTTPPSVYPLAPGSGDTTGSSVTLGCLVKGYFPESVTVTWNSGSLSSSVHTFPALLQSGLYTMSSSVTVPSSTWPSQTVTCSVAHPASSTTVDKKLEPSGHHHHHH

1. Light chain of 1-arm IgG

DIVMTQSPAIMSASPGEKVTMTCSTNSSISYMHWYQQKPGTSPKRWIYDTSKLASGVPARFSGSGSGTSYSLTISSMEAEDAATYYCHQRSSYPWTFGGGTKLEIKRADAAPTVSIFPPSSEQLTSGGASVVCFLNNFYPKDINVKWKIDGSERQNGVLNSWTDQDSKDSTYSMSSTLTLTKDEYERHNSYTCEATHKTSTSPIVKSFNRNE
